# Detection and Location of Surface Damage Using Third-Order Combined Harmonic Waves Generated by Non-Collinear Ultrasonic Waves Mixing

**DOI:** 10.3390/s21186027

**Published:** 2021-09-09

**Authors:** Weibin Li, Tianze Shi, Xiaoxu Qin, Mingxi Deng

**Affiliations:** 1 School of Aerospace Engineering, Xiamen University, Xiamen 361005, China; liweibin@xmu.edu.cn (W.L.); 35120191151185@stu.xmu.edu.cn (T.S.); shiki2010@163.com (X.Q.); 2 Xiamen Engineering Technology Center for Intelligent Maintenance of Infrastructures, Xiamen 361005, China; 3 College of Aerospace Engineering, Chongqing University, Chongqing 400044, China

**Keywords:** nonlinearity, non-collinear mixing, fatigue damage, surface corrosion

## Abstract

Metals which are widely used in many types of industries are usually subjected to fatigue and surface corrosion. There is a demand to detect the surface damage caused by fatigue and corrosion at an early stage to ensure the structural integrity. In this paper, a novel nonlinear ultrasonic technique based on the measure of third-order combined harmonic generation, is proposed to detect and locate the surface damage in 6061 aluminum alloy. Third-order combined harmonic generation caused by non-collinear mixing of one longitudinal wave and one transverse wave at different frequencies, is firstly analyzed and experimentally observed. An experimental procedure of nonlinear scanning is proposed for the damage detection and location by checking the variation of frequency nonlinear response. The correlations of nonlinear frequency mixing responses and surface damage in the specimens are obtained. Results show that the nonlinear response caused by fatigue damage and surface corrosion can be identified and located by this method. In addition, this approach can exclude the nonlinearity induced by the instruments and simplify the signal processing.

## 1. Introduction

Metallic materials are widely used in various fields such as construction, manufacture, and aerospace applications. During their service process, fatigue damage and surface corrosion are the two main factors to cause the degradation of structure integrity, which may ultimately lead to a sudden catastrophic failure [1]. Ultrasonic nondestructive testing (NDT) techniques have been considered as the feasible methods for damage detection. Linear ultrasonic techniques, which are based on the velocity and attenuation, however, are not adequately sensitive to early damage detection [2,3]. Nonlinear ultrasonic testing based on the second harmonics generation (SHG) has been proposed as a potential method for the detection of micro-defects [4,5,6,7,8]. When it comes to surface damages, the use of nonlinear Rayleigh surface wave has been applied to assess the micro-surface damage in metallic materials [9,10,11]. However, there are two inevitable disadvantages for the use of SHG based nonlinear ultrasonic technique, including the uncertain nonlinear sources either from instruments or material, and it is hard to locate the local damage by the SHG of ultrasonic waves [12,13,14].

Nonlinear ultrasonic technique based on non-collinear bulk waves mixing has the potential to overcome these drawbacks. Compared with SHG, the non-collinear technique allows the great flexibility in localizing the position of damage [15,16]. This technique enables the scanning of damaged structures by controlling the time delays of incident signal, frequencies, and the positions of transducers, to thus determine the interaction area for the appearance of nonlinear response. Sun et al. investigated the scanning non-collinear wave mixing for nonlinear ultrasonic detection and localization of plasticity [17], the results indicated that the non-collinear wave mixing is sensitive to the plasticity with an even, small, plastic strain. Croxford et al. reported the use of non-collinear mixing for nonlinear ultrasonic detection of plasticity and fatigue, in which a nonlinear parameter related to the amplitudes of the exciting signals and generated signals was proposed to evaluate the degree of damage. Their results show that the non-collinear mixing technique is capable to measure the changes in both plastic deformation and low-cycle fatigue [18]. Earlier reports are mainly about the investigations of second-order combined harmonic wave generation caused by the ultrasonic wave mixing, while the use of third-order combined harmonic generation has been rarely employed for nondestructive testing. In fact, only in the case where the material nonlinearity due to atomic nonlinearity or at the atomic scale is considered, has the magnitude of second-order combined harmonic been bigger than that of third ones. However, damage induced nonlinearity is complicated, whereas the nonlinear mesocopic elasticity, which is also called classical nonlinearity, is insufficient to describe material nonlinearities in damaged materials well [19], which requires us to be more flexible in choosing the acoustic nonlinear response to quantify the damage.

In this paper, nonlinear frequency mixing of two ultrasonic waves with different frequencies is investigated to distinguish the newly generated mixing signals from the higher order harmonics. Firstly, the theoretical fundamentals of wave mixing are briefly addressed. An experimental setup is proposed to measure the nonlinear frequency mixing response in an intact specimen. Instead of the measure of second-order combined harmonic waves, this paper focuses on the measure of third-order combined harmonic waves caused by the mixing of one longitudinal wave and one transverse wave. The proposed procedure is applied in the surface corrosion and surface fatigue crack detection. The appropriate nonlinear mixing response was employed to represent the acoustic nonlinear parameter. The correlation of acoustic nonlinear parameter and surface damages were obtained. The variations of acoustic nonlinear parameter via nonlinear scanning in the specimens are used to locate the local surface damage.

## 2. Fundamental Theory

### 2.1. Principle of Ultrasonic Waves Mixing

In the case of two ultrasonic waves mixing in an intact specimen without material nonlinearity, there is no mutual interactions of these two waves, thus no nonlinear frequency mixing response will be generated. On the other hand, these two waves meet at the place with material nonlinearity (due to cracks or other damages), other nonlinear frequency components will be generated. The measured nonlinear frequency mixing response can be used to assess the damage level. Mixing of these two primary waves will generate a third propagating wave at either the sum– or difference–frequency. The amplitude of the third wave is a function of the nonlinear parameter of the material in the mixing area. Thus, the new generated wave will provide useful information sensitive to the local nonlinear changes in the structure.

In general, all types of ultrasonic waves can be used for nonlinear mixing testing under the resonant condition. According to the propagation direction of the incident waves, the techniques based on the measure of nonlinear frequency mixing response can be divided into two types, i.e., the collinear and non-collinear wave mixing. Collinear mixing commonly uses two ultrasonic waves propagate in a line with same direction, while more options are available for non-collinear wave mixing, such as different combinations of longitudinal wave and transverse wave. Jones gave a detailed derivation of the wave types and propagation directions of the mixing signals corresponding to various combinations [20]: the interaction of two longitudinal waves can produce the difference–frequency transverse wave signal of the incident waves. The interaction of two transverse waves can produce the sum–frequency longitudinal wave signal of the incident waves. When a combination of transverse wave and longitudinal wave is excited, the mixed signal will contain both the sum–frequency longitudinal wave and the difference–frequency transverse wave.

For the case of third-order combined harmonic generation caused by the interaction of one longitudinal wave and one transverse wave, the resonant third-order combined waves can be generated only if the synchronism conditions are satisfied, which can be described as:

(1) $$k_{3}=k_{1}\pm 2k_{2},$$

(2) $$f_{3}=f_{1}\pm 2f_{2},$$

where $k_{1}$, $k_{2}$, and $k_{3}$ are the wave vectors of two input waves and generated third-order combined harmonic wave, respectively, and $f_{1}$, $f_{2}$, and $f_{3}$ are the frequencies of two primary waves and the corresponding nonlinear mixing frequency, respectively.

### 2.2. Principle of Ultrasonic Waves Mixing

Based on the previous analysis [16], it has been shown that odd harmonics generation is a more general nonlinear behavior of acoustic waves in damaged material with hysteretic nonlinearity. Analogously, the ultrasonic wave mixing, that involving waves at frequency of $f_{1}$ and $f_{2}$ would result in the third-combined harmonic generation at frequency of $2f_{1}\pm f_{2}$ or $f_{1}\pm 2f_{2}$. It is also known that the nonlinear frequency mixing response can only be effectively measured under the resonant condition [21,22,23], in the case of one transverse wave $\left(f_{1}\right)$ and one longitudinal wave $\left(f_{2}\right)$ mixing, while the sum–frequency signal of the generated second harmonic of transverse wave and the fundamental frequency longitudinal wave ($2f_{1}+f_{2}$), and the sum–frequency signal of the generated second harmonic of longitudinal wave and the fundamental frequency transverse wave ($f_{1}+2f_{2}$), which are caused by damage can be effectively detected, even without satisficing the resonant condition. Thus, the third-order combined harmonics at sum–frequency, which are caused by the longitudinal wave and transverse wave mixing is used to detect the damage. Relative nonlinear parameters to characterize the nonlinear response is given by:

(3) $$\begin{array}{l} \beta_{1}=\frac{A_{3}}{A_{1}{}^{2}A_{2}},\\ \beta_{2}=\frac{A_{4}}{A_{1}A_{2}{}^{2}}, \end{array}$$

where *A*_1_ and *A*_2_ are the amplitudes of two primary waves, and *A*_3_ and *A*_4_ are the amplitude of the third-order combined harmonics at sum–frequency of $2f_{1}+f_{2}$ and $f_{1}+2f_{2}$ respectively.

### 2.3. Design of Wedge Angle

**Figure 1:**
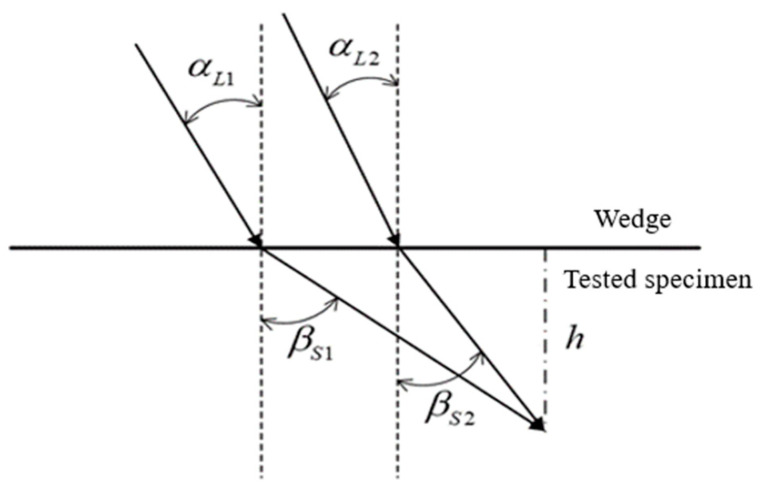
Comparison of different wedge angles with the same incident depth.

When a longitudinal wave is obliquely incident at the interface of two different solid mediums, a refracted longitudinal wave will be generated. A refracted transverse wave will also be generated simultaneously. Longitudinal wave refraction angle is greater than that of transverse wave. In order to exclude the interference caused by the refracted longitudinal wave, an appropriate wedge angle needs to be calculated to ensure a total reflection of the longitudinal wave. According to Snell’s law, the wedge angle should be between the first and second critical angles, i.e., the following formula is satisfied

(4) $$\arcsin\left(c_{L1}/c_{L2}\right)<\alpha <\arcsin\;\left(c_{L1}/c_{S2}\right),$$

where $c_{L1}$ and $c_{L2}$ are the longitudinal wave velocities in material 1 (wedge) and material 2 (tested specimen), respectively, and $c_{S2}$ is the transverse wave velocity in material 2. The angle of the wedge is supposed to be between 26.3° and 61.4° when the longitudinal wave velocity in wedge of plexiglass and the longitudinal wave and transverse wave velocity in 6061. It is important to note that the smaller wedge angle is corresponding to the shorter distance in the horizontal direction, in the case of same detection depth is the same as shown in Figure 1.

**Figure 2:**
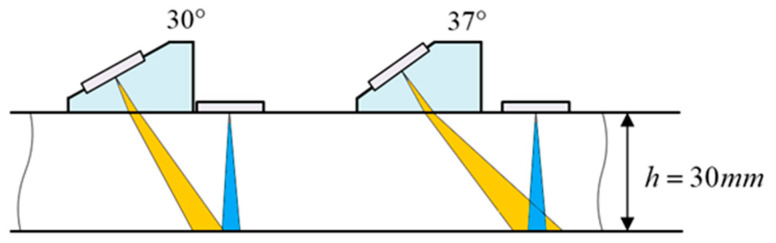
Schematic of 30° and 37° angle beam transducer sound fields.

As another longitudinal wave transducer needs to be placed beside the wedge, there is a limit distance between the wedge and the longitudinal wave probe for the full-interactions and mixing of two excitation signals happened on the surface of tested specimen. The acoustic fields of two incident signals are simulated using software (Beam Tool) to predict the mixing zone. As shown in Figure 2, it can be seen that the 30° wedge is not suitable, as the sound fields of two waves are not well overlapped on the surface of tested specimen, while the 37° wedge can be met the requirement for the experimental test.

### 2.4. Set of Time Daley

**Figure 3:**
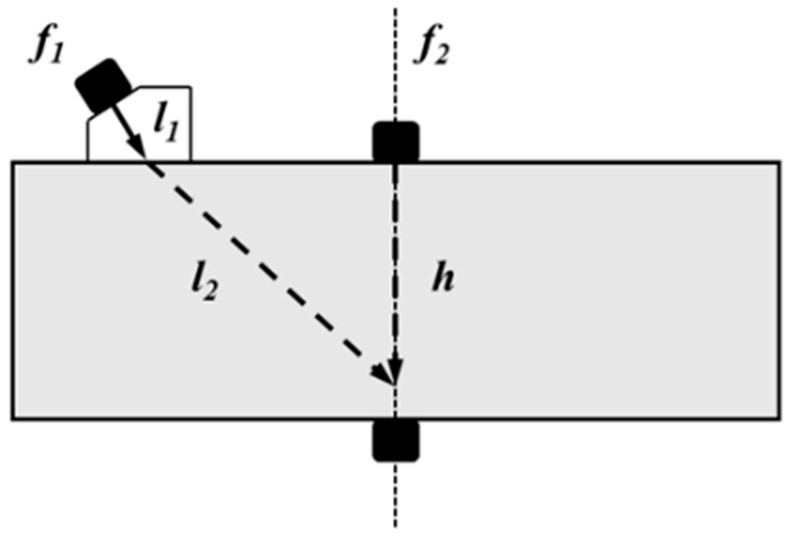
Model used to for the non-collinear wave mixing technique.

According to Snell’s law, the angle of the transverse wave propagates in the sample can be obtained, and the wave path of the signal is calculated to determine the time delay of incident signal for the generation of longitudinal wave. Figure 3 shows the propagation of the signals in the sample, where *l*_1_ is the distance traveled by the longitudinal wave in the wedge, *l*_2_ is the distance traveled by the refracted transverse wave in the specimen, and *h* is the distance traveled by the longitudinal wave in the sample. The propagation time can be calculated as shown in Table 1 according to the wave velocity corresponding to each propagation distance.

The time delay of the 2 MHz excitation signal is 2 μs. Therefore, the theoretical propagating time for the transverse wave from the incident place to the receiving transducer is 2 + 4.41 + 13.2 = 19.61 μs. As the propagation time of the longitudinal wave in the sample is $h/c_{L}=4.87\;\mu s$, the delay of the longitudinal wave is supposed to be 19.61 − 4.87 = 14.74 μs. The distance between the center of the longitudinal wave transducer and the center of the wedge is 2.8 cm.

## 3. Specimens and Experimental Setup

### 3.1. Specimens

The samples used in this study are 6061 aluminum alloy specimens. Two types of specimens were prepared for the measurements with non-collinear wave mixing technique: specimens with corrosion and fatigue crack on the surface, respectively. The dimensions of the corrosion specimens are 30 mm × 60 mm × 300 mm. The dimensions of the fatigue cracking specimens are 53 mm × 60 mm × 280 mm. The fatigue crack is made in the middle of the specimens by the INSTRON-8801 fatigue testing machine. The length of the fatigue crack is 2 mm. The length of the fatigue crack is 2 mm. The corrosion specimen is made by corroding a 25 mm × 25 mm area for 60 s on the surface with Keller reagent and pure hydrochloric acid.

### 3.2. Experimental Setup

This experiment setup is developed to detect and locate two types of surface damages on 6061 aluminum alloy. As the mixing waves provide us with the material information of the intersection area, it is only necessary to control the incident waves mixing zone in the damage area, and then measure the frequency mixing response of received signal to analyzed the information about the surface damage of the specimen.

If the frequencies of two incident waves are the same, the received time–domain signal needs to be processed in order to distinguish the mixing signal from the higher order harmonics. To avoid this problem, two incident waves with different frequencies are used in this experiment. In that case, the sum–frequency signal of the two excitation signals and their second harmonics can be well separated from each other.

**Figure 4:**
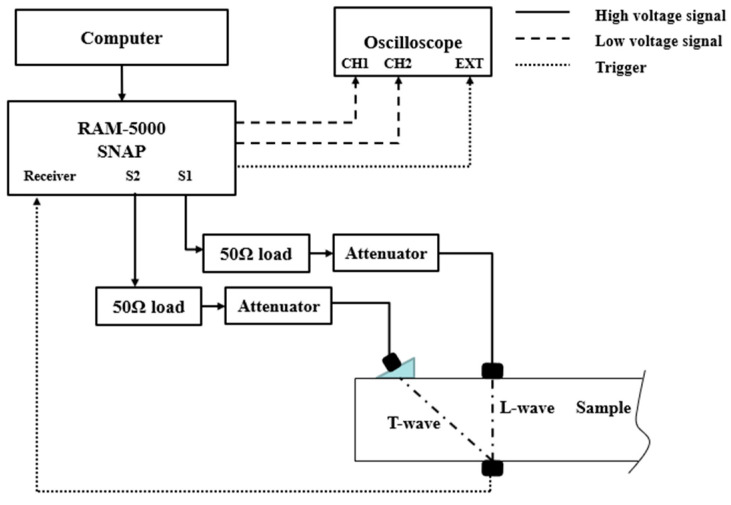
Schematic diagram of the experimental system.

Collinear mixing can be achieved with just two transducers, but one of the transducers needs to cooperate with the duplexer, thus requiring it to have a wider frequency band. The wedge transducer, consisting of the plexiglas wedge (with an oblique angle) and the ultrasonic transducer at central frequency of 2.25 MHz (A188S-RB, Panametrics Inc., Waltham, MA, USA), is used to generate the desired transverse wave, while the contact longitudinal wave transducer at central frequency of 1.0 MHz (A539S-SM, Panametrics Inc.), is used to generate the desired longitudinal wave. A contact transducer with central frequency of 5.0 MHz (A108S-RB, Panametrics Inc.) is used to measure the third-order combined harmonic waves. Additionally, the method of collinear mixing will lead to the overlap of excitation signal and received signal, which is a drawback for the signal processing. Therefore, the non-collinear wave mixing method is adopted in this study. A longitudinal wave is excited vertically, and a transverse wave is excited obliquely through the longitudinal wave probe and an angle wedge. The mixed signal is received on the other side of the sample. A schematic diagram of the non-collinear wave mixing experimental system is shown in Figure 4.

The high voltage output of the RITEC SNAP 5000 nonlinear ultrasound system produces two signals of different frequencies through high power gated amplifiers. Channel 1 (S1) is used to generate a 5 μs longitudinal wave tone-burst, while the transverse wave tone-burst is generated by channel 2 (S2) and passes through a wedge at 37° with the same duration. Both of the generated sinusoidal tone-burst signals pass through the attenuator, which is set to purify the incident signal for a high signal-to-noise ratio (SNR). One narrow band piezoelectric transducer, whose central frequency is 1.0 MHz, is used to excite the longitudinal wave (L-wave), and another narrow band piezoelectric transducer, whose central frequency is 2.25 MHz with the designed wedge, is used to excite the transverse wave (T-wave). As the longitudinal wave travels faster than the transverse wave, a pulse delay of 14.74 is set up for the longitudinal wave signal in order to control the two waves reaching the other surface simultaneously. The two signals interact in the mixing zone on the surface, theoretically producing the sum–frequency, difference–frequency, and high order harmonics signals of the fundamental frequency waves. As the interaction occurs on the bottom surface of the sample, the mixed signals can be received by the receiving transducer, a broadband piezoelectric longitudinal wave transducer who has a central frequency of 5.0 MHz, placed beneath the specimen.

## 4. Results and Discussion

### 4.1. Non-Collinear Mixing Tests in Intact Samples

#### 4.1.1. Mixing of 2.0 MHz Transverse Wave and 1.0 MHz Longitudinal Wave

As analyzed in Section 2, it is critical to select the incident bulk waves which satisfy the synchronism conditions for non-collinear wave mixing technique. The first setup is to verify that the signal detected is the desired waves at determined frequencies. Three measurements with different excitations are conducted on each specimen:

- Only the transverse wave is generated;
- Only the longitudinal wave is generated;
- Both of these two bulk waves are generated.

**Figure 5:**
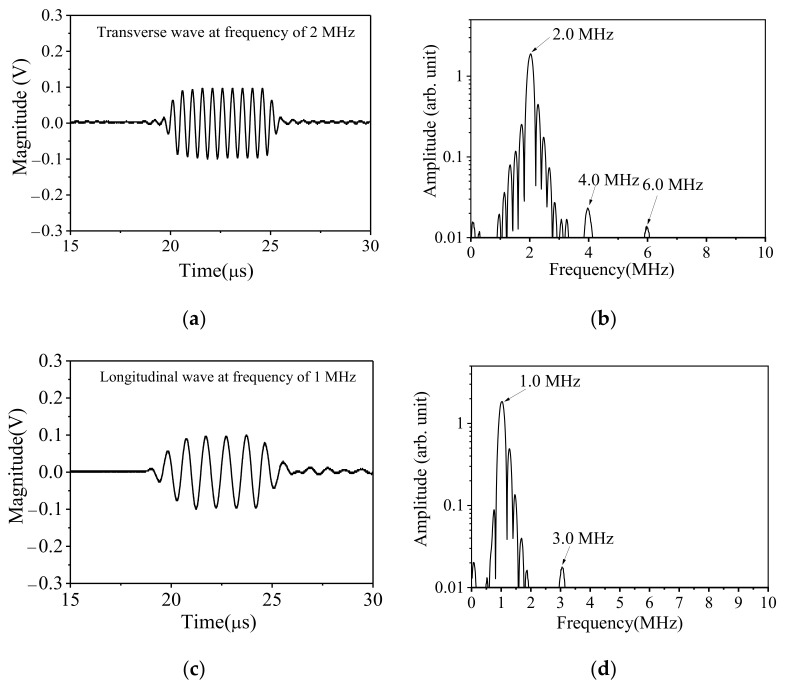
Time–domain signals (**a**,**c**) and amplitude–frequency curves (**b**,**d**) of the transverse wave and longitudinal wave generated individually.

The time– and frequency–domain diagrams of the received signals of both incident waves are shown in Figure 5, where the central frequencies of the received signals are 2.0 MHz and 1.0 MHz, respectively. Furthermore, the time of signal receiving is very close to the theoretical value. Thus, we are convinced that these two signals are the desired waves.

In addition, when the 2.0 MHz signal is excited individually, there are the 4.0 MHz and 6.0 MHz high-order harmonics in the received signal except for the fundamental frequency signal, though they are not obvious. When the 1.0 MHz signal is individually excited, there are weak third-order harmonics in addition to the fundamental frequency components in the received signal, and the second-order harmonics are not obvious either.

**Figure 6:**
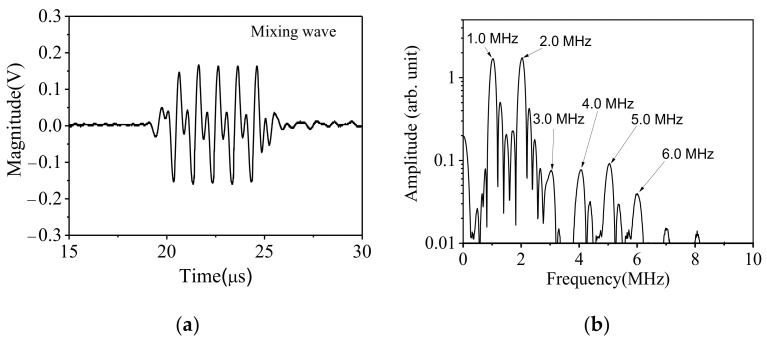
(**a**) time–domain signal received when two primary waves are excited simultaneously and (**b**) the corresponding amplitude–frequency curve.

Figure 6a shows the time–domain response when the two bulk waves intersect and interact at the desired region. Compared with the 2.0 MHz and 1.0 MHz signal excited separately, a significant distortion occurs to the mixing signal’s waveform. However, it is still necessary to confirm the presence of a new frequency component by further performing a Fast Fourier Transform (FFT) on the received signal, and the result is shown in Figure 6b.

As the nonlinear ultrasonic detection system (RAM 5000 SNAP) used in the experiment has only two channels (S1 and S2), which are used to excite the two incident signals of different frequencies, therefore, the excitation signals cannot be window modulated. As can be seen from Figure 6b, there are some unavoidable sidebands in the frequency–domain. Compared with the frequency–domain signal in Figure 5, when the two signals are excited at the same time, the received signal obviously contains newly generated mixing waves, which are mainly the signals of 3.0 MHz, 4.0 MHz, and 5.0 MHz, except for the 6.0 MHz and 7.0 MHz signals.

In addition to the incident fundamental–frequency signal and their high order harmonics, the mixing signal has new components, which indicates that the two incident signals have a nonlinear interaction in the near surface area of the specimen. This confirms the feasibility of the idea of non-collinear mixing with two waves of different frequencies. Compared with the conventional method of mixing signals in a certain area inside the sample and receiving the sum–frequency or difference–frequency mixing waves in a specific direction, this method provides more choice for the construction of the ultrasonic nonlinear parameter.

Although the amplitudes of 3.0 MHz and 4.0 MHz signals are much higher than that when the fundamental frequency signals are excited individually, they cannot be completely attributed to the interaction of the two input signals as they are overlapped with the third order harmonics of the 1.0 MHz signal and the second order harmonics of 2.0 MHz signal. The 5.0 MHz signal does not show up when the fundamental frequency signals are excited individually, but it may come from the interaction of the third harmonic of 1.0 MHz signal and the 2.0 MHz fundamental signal, or the interaction of the second harmonic of 2.0 MHz signal and the 1.0 MHz fundamental signal. Therefore, it is necessary to adjust the frequency of the excitation signals and perform the mixing experiment again.

#### 4.1.2. Mixing of 2.0 MHz Transverse Wave and 1.1 MHz Longitudinal Wave

**Figure 7:**
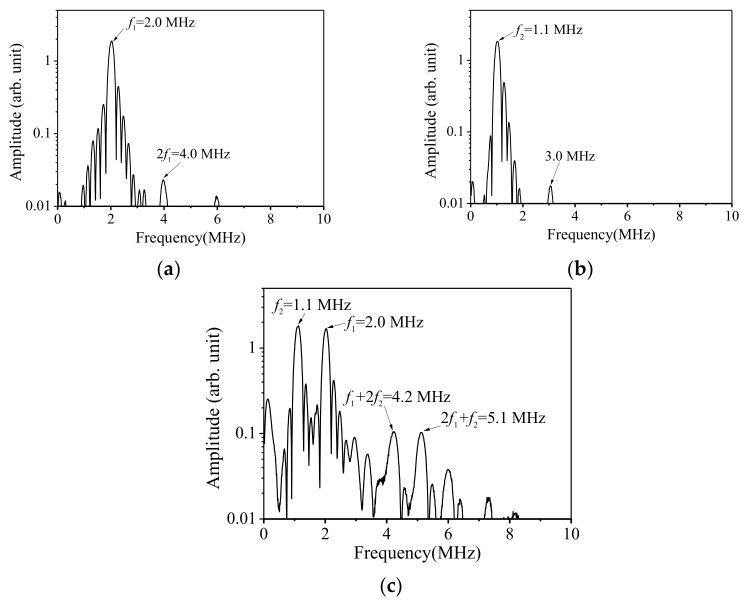
Amplitude–frequency curves when the 2.0 MHz transverse wave is individually excited (**a**), the 1.1 MHz longitudinal wave is individually excited (**b**), and both these two primary waves are excited simultaneously (**c**).

The frequency of the longitudinal wave signal is increased to 1.1 MHz while the frequency of the transverse wave is kept constant for 2.0 MHz, and the above experiment is repeated. The FFT is applied to the received signals again, and the frequency–domain curves are shown in Figure 7.

Compared with the previous results of mixing 2.0 MHz transverse wave and 1.0 MHz longitudinal wave, the frequency domains of 2.0 MHz transverse wave and 1.1 MHz longitudinal wave have not changed a lot, while the frequency domain of the mixed wave has obviously changed. The following characteristics are observed through comparison:

- When the two signals of 2 MHz and 1.1 MHz are excited simultaneously, there is no obvious sum–frequency signal (3.1 MHz) in the frequency domain.
- Third-order combined harmonic at frequencies of 4.2 MHz and 5.1 MHz clearly appear.
- The peak of the 6.0 MHz signal does not shift.

The phenomenon of no second-order combined harmonic waves generation at sum–frequency, can be attributed to the domination of odd harmonic generation caused by damage induced nonlinearity. The second feature solves the previous question, i.e., where the 5.0 MHz signal comes from. By analysis, we can confirm that the 5.1 MHz signal is the sum–frequency signal from the second harmonic of the 2.0 MHz signal and the 1.1 MHz fundamental signal. In addition, the 4.0 MHz signal peak has shifted to 4.2 MHz, which indicates that the 4.2 MHz signal is the sum–frequency signal from the second harmonic of the 1.1 MHz signal and the 2.0 MHz fundamental signal.

On the third point, the 6.0 MHz signal peak does not shift, indicating that the signal mainly comes from the third harmonic of the 2.0 MHz signal, regardless of the frequency of the longitudinal wave. Additionally, its peak value significantly increases compared with the frequency domain of the received signal when the 2.0 MHz signal is excited individually. The reason may be that the incidence of the 1.0 MHz/1.1 MHz longitudinal wave signal plays a modulating role, which amplifies the nonlinear response in the material.

It is important to note that the phenomenon of third-order combined harmonic generations rather than second-order ones has unique advantage in this study, which can be used to indicate that the nonlinear frequency mixing response observed is induced by the non-collinear cross-interaction of the longitudinal and transverse waves in the specimen. In other words, if the third-order combined harmonics are attributed to the experimental instruments, the second-order combined harmonics will be inevitably generated due to the second-order cross-interaction between them, and generally the magnitude of the second-order combined ones will be larger than that of the third-order ones. The domination of the third-order combined harmonic waves, which are experimentally observed in this study, can not only shows that the synchronism condition is necessary for generation of the combined harmonics, but also indicates that the frequency mixing response measured is mainly due to the damage induced nonlinearity in specimen, rather than that from the instrumental system.

### 4.2. Detection and Location of Fatigue Damage by Non-Collinear Wave Mixing

According to the theory of nonlinear wave mixing, the acoustic nonlinearity is only generated when the two primary waves intersect and interact in the region associated with nonlinearity. Therefore, by changing the positions of mixing zone, the spatial distribution of acoustic nonlinear parameter can be obtained. To suppress the variations of acoustic nonlinearity caused by coupling effect when moving the transducer, a baseline measurement in the initial position of the specimens is made. Only the measured signals of nearly the same magnitude at different positions, are used to calculate the nonlinear parameters to alleviate the experimental uncertainly [12].

In this section, the fatigue cracking specimen and corrosion specimen are scanned using the above experimental system, and the surface damages are evaluated by the acoustic nonlinear parameter *β* so that the defects can be located.

#### 4.2.1. Corrosion Sample Scanning

**Figure 8:**
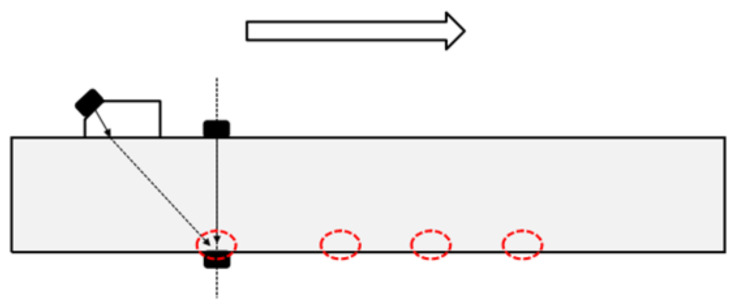
Geometry of the model used to scan the surface of the specimen.

The length of the specimen is 300 mm; 5.0 cm away from the left edge of the sample, nine positions to be measured are selected every 2.5 cm. All the positions are located on the central axis of the specimen surface. The scanning process is shown in Figure 8.

**Figure 9:**
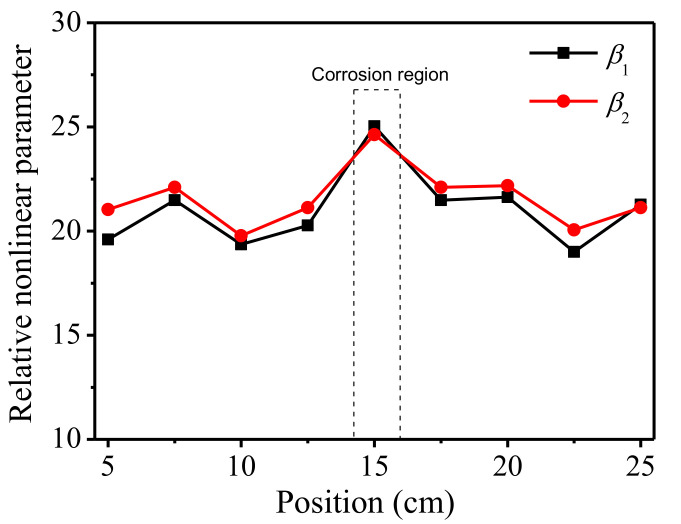
Nonlinear response at different locations of the specimen.

The received time–domain signals are processed into frequency–domain curves using FFT. The amplitudes are measured, and the acoustic nonlinear parameters are calculated according to Equation (3). It can be seen from Figure 9 that the acoustic nonlinear responses vary slightly at different locations on the surface of the specimen. The trends of the two nonlinear parameters constructed in this paper are very similar. In particular, the acoustic nonlinear response at the 15 cm position where the corrosion defect is located is slightly higher than the other positions.

The correlation coefficient of the nonlinear response parameters *β*_1_ and *β*_2_ is 0.967, which proves that the two nonlinear parameters constructed in this paper are related to one another. *β*_1_ increases by 22.0% and *β*_2_ increases by 16.2% in the corroded area compared with the average of the nonlinear responses measured in other positions. This means that the nonlinear ultrasonic detection method of non-collinear mixing of two bulk waves can locate the corrosion defects to a certain extent.

#### 4.2.2. Fatigue Sample Scanning

**Figure 10:**
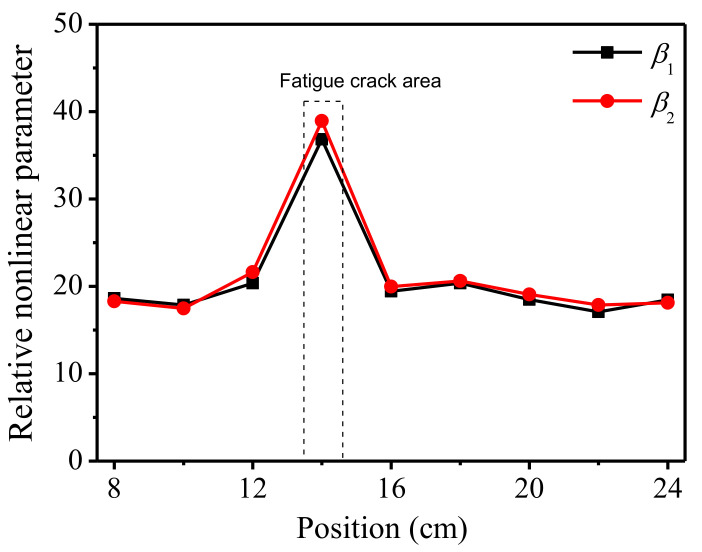
Nonlinear response at different locations of the fatigue specimen.

As the thickness of the fatigue sample is larger than that of the corrosion one, the wave path of the transverse wave in the sample is longer and, accordingly, the distance between the longitudinal wave probe and the wedge is greater. Therefore, when the sample is scanned horizontally, the range of movement of the probes is smaller than that of the corrosion sample. The scanning starts at a distance of 8 cm from the left edge of the specimen, measuring once every 2 cm until reach the distance of 4 cm from the right edge, a total of nine positions. The positions are located on the center axis of the specimen surface. The relative nonlinear parameters at different positions are shown in Figure 10.

As can be seen in Figure 10, compared with other positions, the mixing response significantly increases by nearly 100% in the fatigue crack area (14 cm). Therefore, the bulk wave non-collinear mixing technique can be a promising tool to detect and locate fatigue damages on the surface of structures. Incidentally, the nonlinear response of the no-defect positions is slightly lower than that of the corrosion one, which may be caused by milling the fatigue specimen.

Variations of acoustic nonlinear response at different locations of the sample are used to identify the local damage. As shown in Figure 9 and Figure 10, it can be found that the sharp increase in nonlinear parameters at certain position of the specimen, can be used to identify and locate the damage. On the other hand, the nonlinear parameters are consistent and stable in the intact region of the specimen.

It is noticed that the acoustic nonlinear parameters measured by proposed approach are more sensitive to fatigue crack than corrosion. The objective of this investigation is to provide an alternative method to detect and locate the surface damage nondestructively. Identification of various types of corrosions is beyond the scope of this investigation. The results of the experiment confirm the effectiveness of the wave mixing nonlinear ultrasonic parameters constructed in this paper. Comparing the scanning results of two types of damaged specimens, it is found that the non-collinear mixing response with bulk waves is more sensitive to fatigue cracking. The reason may be that the two types of damage can cause different amounts of nonlinear response, or the size of damaged area of the two specimens is different from one another. It is shown that the intersection area of the two signals is an overlapped part of the two cones, which occupies a certain space. Therefore, the validity of the method for locating the surface defects is confirmed by comparing the acoustic nonlinear parameters at different positions with and without two types of surface defects including fatigue crack and corrosion.

## 5. Conclusions

In this work, two bulk waves with different frequencies are used for the detection of corrosion and fatigue damage at the surface by non-collinear wave mixing technique. Nonlinear frequency response of one longitudinal wave and one transverse wave at different frequencies, mixing collinearly, is measured to characterize the surface damage in 6061 aluminum alloy. An experimental procedure of nonlinear scanning is proposed for the damage detection and location by checking the variation of frequency nonlinear response. The specimens are scanned by the proposed wave mixing method. The validity of the method for locating the surface defects is confirmed by comparing the acoustic nonlinear parameters at different positions. It is shown that the non-collinear wave mixing technique can be a promising tool for the detection of fatigue damage location in 6061 aluminum alloy.

## Figures and Tables

**Table 1:** Calculation of transverse wave propagation time.

Section	Distance (mm)	Velocity (m/s)	Propagation Time (μs)
*l_1_*	12.0	2720	4.41
*l_2_*	41.0	3097	13.2

## Data Availability

The data presented in this study are available on request from the corresponding author.
